# INDIAN JOURNAL OF DERMATOLOGY GETS RE-INDEXED WITH PUBMED

**DOI:** 10.4103/0019-5154.60341

**Published:** 2010

**Authors:** Sandipan Dhar

**Affiliations:** *IJD E-mail: drsandipan@gmail.com*

The year 2009, the 54^th^ year of the journal, will be a landmark in the history of IJD. After 19 years of its de-indexed state, the journal finally entered the PubMed Central (PMC) on October 30, 2009, and got indexed with PubMed on November 5, 2009. Issues from 2008 are linked to the PubMed site and the citations are available.

It may be worth mentioning here that it will be practically impossible to draw a parallel to the Indian Journal of Dermatology either in the country or anywhere else in the world where a scientific journal (not only in dermatology but in the entire field of biomedical journals) published by a state branch of a society could not only survive for such a long period but also got itself indexed in PubMed. In 2009, we had a record 331 submissions, of which 63 were original articles. The policy of the journal is to invite Review and CME articles from academicians who have worked and contributed significantly in a particular field. However, we have been overwhelmed by the growing number of good quality review articles being uploaded to the IJD website by various contributors. This spontaneous and enthusiastic response of our colleagues has encouraged us a lot in our efforts to make the journal better and richer in its content.

The current acceptance rate of the journal is six per cent. The figure is comparable with some of the most prestigious journals in the field. Presently it takes about six to eight months for an original contribution and 12 to 18 months for a case report to be published. We have been working hard to shorten this period.

We have introduced a new section named ‘Symposium in Dermatology’ from the April-June'09 issue onwards. Our objective is to showcase articles on issues of particular relevance to the students, clinicians and researchers of dermatology in this subcontinent, though we shall welcome articles with a still wider appeal. We have already published symposia on ‘Vitiligo Surgery’ and ‘Urticaria’ which have received a lot of appreciation from our readers. We hope to continue it in future regularly on a wider range of subjects. We have published a good number of scholarly articles by some of the most eminent leaders in dermatology of the world, this year.

Another important achievement of the journal in 2009 has been its worldwide popularity. Several articles from IJD had featured regularly in the the MDLinx site which is supposed to be the most read site by US physicians. Some of the articles have even been placed at the top position. This is a phenomenal event as hardly any Indian journal in any specialty has been found to feature in the site as frequently as IJD. The strength of IJD has been its rich content. We have regularly published ‘Basic Research’ articles over the last three years and we have also published good number of original articles on various important issues.

I am privileged to thank my two most able colleagues, both executive editors, Koushik Lahiri and Saumya Panda, for their tireless efforts and dedicated contributions. I take this opportunity to thank Tapas Kayal, my assistant, for smooth execution of the job allocated to him. I also thank all my hard working editorial team members, the committed editorial and editorial advisory board members, sincere reviewers, erudite contributors and, above all, our selfless unbiased readers.

I take this opportunity to thank all the esteemed executive body members of IADVL, WB branch over last four years for support and co-operation during my tenure. I must acknowledge and thank Dr. D K Sahu of Medknow Publications for his help, support and guidance to get the journal indexed. His house has not only been the publisher for the journal but has also given very important and useful inputs regarding improving the quality of the journal.

A journal can never run smoothly without funding from the pharmaceutical industry. I hereby thank Galderma India Pvt.Ltd, Fulford India Ltd, Glenmark Pharmaceuticals, Palsons Drugs and Chemical Industries, Dr. Reddy's Laboratories, Janssen-Cilag Pharmaceuticals, Sanofi-Aventis Pharma, who have supplied the necessary oxygen for the journal's survival.

As was written in the Editor's epistle “…at this moment of pride and joy, the Editorial team feels redeemed for being able to stand true to the ideals and vision of the founding fathers of the journal, able to nurture and nourish it like a delicate plant that has its roots firmly on the ground beneath, yet has an insatiable urge to reach out to the sky, to the wide world far beyond immediate environs. This great moment asks all of us in the Editorial Team to be humble and introspective, humble in the realization that this historic opportunity demands us to take the journal to ever greater heights, introspect all the time as to how much further we need to carry forward our unfinished task…”. However sweeping the changes might seem, our basic objective remains the same: ‘to be devoted to the advancement of the subject’ i.e., dermatology.

I, on behalf of ‘Team IJD’, sincerely thank all of you for your kind patronage of IJD and hope you will continue to do the same in the days to come. I wish all of you a very happy and prosperous 2010.

